# The Cross-Protective Immunity Landscape Among Different SARS-CoV-2 Variant RBDs

**DOI:** 10.3389/fimmu.2022.898520

**Published:** 2022-06-10

**Authors:** Wenqiang Sun, Lihong He, Huicong Lou, Wenhui Fan, Limin Yang, Gong Cheng, Wenjun Liu, Lei Sun

**Affiliations:** ^1^ CAS Key Laboratory of Pathogenic Microbiology and Immunology, Institute of Microbiology, Chinese Academy of Sciences, Beijing, China; ^2^ Shenzhen Bay Laboratory, Institute of Infectious Diseases, Shenzhen, China; ^3^ State Key Laboratory for Conservation and Utilization of Subtropical Agro-Bioresources & Laboratory of Animal Infectious Diseases, College of Animal Sciences and Veterinary Medicine, Guangxi University, Nanning, China; ^4^ Savaid Medical School, University of Chinese Academy of Sciences, Beijing, China; ^5^ Tsinghua-Peking Center for Life Sciences, School of Medicine, Tsinghua University, Beijing, China

**Keywords:** SARS-CoV-2, variant, vaccine, RBD, cross-protective immunity

## Abstract

Despite the fact that SARS-CoV-2 vaccines have been available in most parts of the world, the epidemic status remains grim with new variants emerging and escaping the immune protection of existing vaccines. Therefore, the development of more effective antigens and evaluation of their cross-protective immunity against different SARS-CoV-2 variants are particularly urgent. In this study, we expressed the wild type (WT), Alpha, Beta, Delta, and Lambda RBD proteins to immunize mice and evaluated their cross-neutralizing activity against different pseudoviruses (WT, Alpha, Beta, Delta, Lambda, and Omicron). All monovalent and pentavalent RBD antigens induced high titers of IgG antibodies against different variant RBD antigens. In contrast, WT RBD antigen-induced antibodies showed a lower neutralizing activity against Beta, Delta, Lambda, and Omicron pseudoviruses compared to neutralization against itself. Interestingly, Beta RBD antigen and multivalent antigen induced broader cross-neutralization antibodies than other variant RBD antigens. These data provide a reference for vaccine strain selection and universal COVID-19 vaccine design to fight the constant emergence of new SARS-CoV-2 variants.

## Introduction

Since the COVID-19 outbreak, the virus has spread around the world and posed a huge threat to human health (1–4). SARS-COV-2 is an enveloped, unsegmented, single-stranded RNA virus with four structural proteins, namely, E envelope protein, M membrane protein, N nucleoprotein, and S spike protein (5). Among them, S protein contains two subunits, S1 and S2, in which receptor-binding domain (RBD) exists in S1 subunit and is responsible for binding to hACE2 (6–8). Therefore, S and RBD were often selected as targets for SARS-CoV-2 vaccine development (9–12).

Currently, the clinically used SARS-CoV-2 vaccines, including inactivated vaccines, mRNA vaccines, subunit vaccines, adenovirus vector vaccines, and so on, are all based on the wild type (WT) strain (13–20). However, SARS-CoV-2 has been undergoing mutations. Variant Alpha (B.1.1.7), Beta (B.1.351), Gamma (P.1), Iota (B.1.526), Delta (B.1.617.2), Lambda (C.37), and Omicron (B.1.1.529) have been reported successively (21–25). The N501Y mutation was found in RBD of Alpha variant in the UK and has been shown to increase transmissivity and slightly decrease neutralizing activity in recovered patients and immune sera (15, 26, 27). Beta variant was first identified in South Africa, which had three mutations (K417N, E484K, and N501Y) in RBD. These mutations have been reported to affect its binding to human ACE2 and seriously evade neutralizing antibodies (28–31). The Delta variant was first reported in India in 2020 and subsequently became the dominant pandemic strain in the world, where it is still circulating. Two mutation sites (T478K and L452R) exist in Delta RBD. Studies have shown that these two mutations do not participate in its binding to hACE2 (32). Several studies have reported a decrease (mild to moderate) in the neutralization activity of serum of vaccine and recovered patients to Delta variant (33–35). It is possible that the involvement of mutations in other positions cannot be excluded. Lambda variant was first reported in Peru and circulating in parts of Latin America. Two mutation sites (L452Q and F490S) exist in RBD of Lambda. Substantial evidence shows that the Lambda variant exhibits strong immune evasion against vaccines and convalescent sera (33, 36, 37). Omicron was first reported from South Africa in late 2021 and is currently the most dominant strain worldwide. There were 15 mutations in RBD, which affected the receptor-binding motif of RBD interacting with hACE2 (32). The Omicron variant exhibits the most severe immune evasion than other variants (19, 34, 38–40). Therefore, there is an urgent need to develop more effective antigens and evaluate their cross-protective immunity against different SARS-CoV-2 variants.

In this study, we analyzed the genetic evolution and protein structure of different variant RBDs and examined their binding affinity to hACE2. Furthermore, mice were immunized with these variant RBD antigens and the cross-neutralization activity against different variant pseudoviruses were evaluated.

## Materials and Methods

### Cells and Animals

Human embryonic kidney 293T (HEK293T) cells stably expressing hACE2 (293T/hACE2) were kindly provided by Dr. Zhendong Zhao (Institute of Pathogen Biology, Chinese Academy of Medical Sciences & Peking Union Medical College). HEK293T cells (ATCC CRL-3216) were maintained in Dulbecco’s modified Eagle’s medium (DMEM, Gibco) supplemented with 10% fetal bovine serum (FBS, Gibco) and penicillin (100 U/ml)-streptomycin (100 mg/mL) (Thermo Fisher Scientific, Waltham, MA, USA). All cell lines are tested negative for mycoplasma contamination (Mycoplasma Detection Kit, Solarbio, Beijing, China). Female C57BL/6 mice were purchased from Beijing Vital River Animal Technology Co., Ltd. (licensed by Charles River) and housed and bred under 18 ~ 22°C and 50 ~ 60% humidity conditions.

### Construction and Expression of RBD Protein

The different variant RBD proteins were fused with human IgG Fc (hFc) and IL-10 signal peptide, and the amino acid sequences were optimized by human preference codons and constructed into pcDNA3.4 vector. The recombinant plasmid was confirmed by sequencing by Nanjing Genscript Company. Different variant RBD plasmids were transfected into HEK293T cells and the supernatant was harvested 72 h later. Then, the supernatant was purified using Protein A column and proteins were eluted with citric acid buffer (pH 3.4). The eluted proteins were dialyzed in PBS buffer. The purity and molecular weight of proteins were determined by SDS-PAGE. The protein concentration was measured by NanoDrop microspectrophotometer (Thermo Fisher Scientific). Finally, the RBD proteins were frozen to -80°C.

### Affinity Between RBD Proteins and hACE2

Ninety-six well ELISA plates (Corning, USA) were coated with the hACE2 protein (2 μg/mL, SinoBiological) overnight at 4°C and blocked with 4% bull serum albumin. The different variant RBD proteins were diluted to a uniform concentration (1 mg/mL), and then two-fold diluted. The plates were incubated with Goat Anti-Human IgG-HRP (Abcam) and developed by the addition of 100 µL of 3,3’,5’,5-tetramethylbenzidine (TMB) to each well. Finally, 100 µL of 2 mmol/L H_2_SO_4_ was added to terminate the reaction, and the light absorption of the plate was measured at 450 nm using a microplate reader (Thermo Scientific). The fitted curves and EC_50_ were created using Graphpad software (version 8.0). Each experiment was performed three times.

### Mouse Experiments

To evaluate the immune efficacy of different variant RBDs, aged 6-8 weeks naive C57BL/6 mice (four independent mice in each group) were immunized intramuscularly with monovalent and multivalent RBD antigens pre-bound to Alum-adjuvant (antigen and aluminum adjuvant (Imject^®^ Alum, Thermo Scientific) were mixed in a 1:1 volume ratio and gently stirred for 30 min to allow the aluminum adjuvant to effectively adsorb antigen). The immune dose was 10 μg each variant RBD protein per mouse of monovalent RBD antigen and pentavalent RBD antigens were a 10 μg RBD protein mixture including each 2 μg variant RBD protein. Mice immunized with PBS were identified as placebo control. Priming and booster vaccinations were spaced at 3 weeks. Serum samples were collected, inactivated at 56°C for 30 min, and stored at −20°C for subsequent use.

In immunization animal experiments, the clinical status and food intake of the mice were monitored and recorded daily.

### Enzyme Linked Immunosorbent Assay (ELISA)

Ninety-six well ELISA plates (Corning, USA) were coated respectively with six different RBD-hFc protein (1 μg/mL) overnight at 4°C and blocked with 4% bull serum albumin. The serum was threefold diluted and added to each well, and the plates were incubated with Rabbit Anti-Mouse IgG-HRP (Light Chain Specific, CST) and developed by the addition of 100 µL of 3,3’,5’,5-tetramethylbenzidine (TMB) to each well. Finally, 100 µL of 2 mmol/L H_2_SO_4_ was added to terminate the reaction, and the light absorption of the plate was measured at 450 nm using a microplate reader (Thermo Scientific). The endpoint of serum antibody titers was determined as the reciprocal of the highest dilution that was 2.1-fold higher than the optical absorbance value of the negative control. Each experiment was performed three times.

### Pseudovirus Neutralization Assay

For pseudovirus production, the codon-optimized full-length S protein of wild-type (WT) SARS-CoV-2 S protein was cloned in pCAGGS plasmids and the full-length S protein plasmids of Alpha, Beta, Delta, Lambda, and Omicron variants were modified from pCAGGS-WT. The plasmid pCAGGS-S-WT, pCAGGS-S-Alpha, pCAGGS-S-Beta, pCAGGS-S-Delta, pCAGGS-S-Lambda, and pCAGGS-S-Omicron were cotransfected with psPAX2 and pLenti-GFP into HEK293T cells at a mass ratio of 1:1:1. After 72 h, the supernatant containing pseudovirus was harvested and stored at −80°C for subsequent use. The TCID_50_ was determined in 293T/hACE2 cells according to the previous described method (41).

HEK293T/hACE2 cells were inoculated before the experiment. Starting with a 1:10 dilution, each serum sample was continuously diluted twice in a 96-well plate. Equal volumes of WT or variant pseudoviruses were mixed with each diluted serum sample and incubated for 1.5 h at 37°C. The mixture of virus-serum was added to the cells. After 72 h, the cells-supernatant mixture was collected. The firefly luciferase activity in the cells was detected by chemiluminescence, and the luciferase activity was quantified to measure the transduction efficiency. To calculate the neutralization efficiency, a pseudovirus without serum sample was used as a positive control. Each sample was assessed in three repeat wells. Positive values were determined to be relative luminescence unit (RLU) values that were tenfold higher than that of only the cell background. The half-maximum neutralization titer (NT_50_) value was the reciprocal of the dilution of half of the mean RLU value of the positive control.

### Statistical Analysis

All of the data are presented as the mean ± SEM. P-values were determined by one-way ANOVA. All graphs were generated with GraphPad Prism version 8.0 software.

## Results

### Bioinformatics Analysis of SARS-CoV-2 Variant RBD

**Figure 1:**
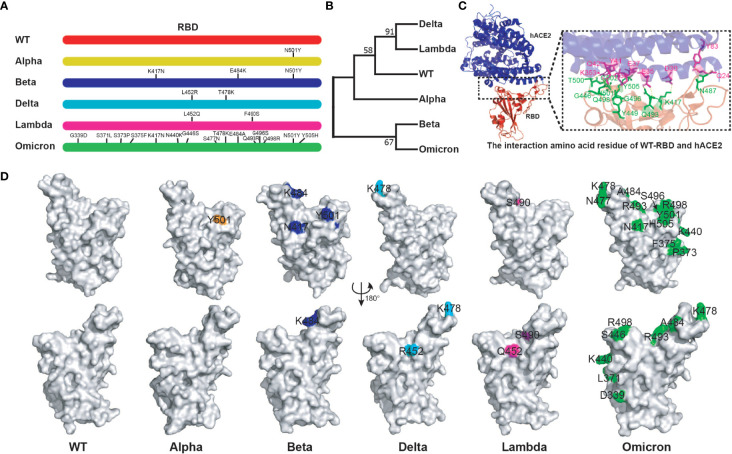
Bioinformatics analysis of SARS-CoV-2 variant RBD. **(A)** The mutation sites of different variant RBDs. **(B)** The evolutionary trees of different variant RBDs. The phylogenetic tree was constructed using the maximum likelihood method within MEGA software (version 11.0). **(C)** The interaction amino acid residues of WT RBD with hACE2 (PDB:6MOJ). Green: RBD amino acid residue; Purple: hACE2 amino acid residue. The image was rendered in PyMOL (version 4.60). **(D)** The distribution of mutant amino acids on RBD structures of different variants. Source of protein crystal structure data from PDB database: WT (6MOJ), Alpha (7EDF), Beta (7PS4), Delta (7WBQ), and Omicron (7WBP). The crystal structure of Lambda RBD was constructed using Swiss-Model online tools. The image was rendered in PyMOL (version 4.60).

The binding of SARS-CoV-2 RBD to hACE2 is crucial to the virus infection. Several key amino acid mutations are found in the RBD of variants (32). We further analyzed the relationship between RBD sequences of different variants and their antigenicity. More striking, there are 15 amino acid mutations in Omicron RBD. Moreover, there were common mutation sites among different RBDs. For example, Beta RBD (K417N, E484K, N501Y) has the same mutation sites as Alpha (N501Y) and Omicron (K417N, N501Y) (**Figure 1A**). Genetic evolution analysis of RBD amino acid sequences of different variants revealed that Delta and Lambda variants formed a cluster, and Beta and Omicron variants formed another cluster (**Figure 1B**), suggesting that the RBDs in the same cluster of phylogenetic trees might have similar antigenicity. Crystal structure analysis of WT RBD and ACE2 complex indicated that the amino acid residues K417, G446, Y449, N487, Q493, G496, Q498, T500, N501, G502, and Y505 were involved in the interaction of virus and host cell receptor (**Figure 1C**). A recent study showed that the amino acid mutations of RBD binding sites to hACE2 have emerged. For instance, Omicron RBD possesses eight substitutions on the hACE2 recognizing interface, namely K417N, G446S, E484A, Q493R, G496S, Q498R, N501Y, and Y505H, compared with what was seen on the WT RBD (32). In addition, the same amino acid substitutions of RBD were found among different variants. N501Y mutation existed in Alpha, Beta, and Omicron variants, K417N was found in Beta and Omicron variants, and T478K was found in Delta and Omicron variants (**Figure 1D**). Importantly, these mutations have been reported to play important roles in host adaptation and immune evasion (42). Together, the above results indicate that the mutation sites among different RBDs might affect their antigenicity, which provides a reference for the selection of vaccines against different variants.

### Increased Affinity Between Variant RBD and hACE2

**Figure 2:**
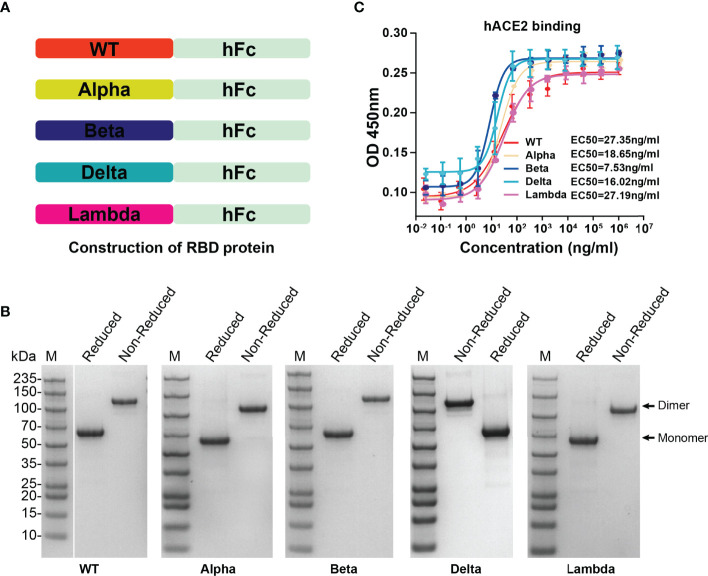
The expression of SARS-CoV-2 variant RBD proteins and determination of affinity between variant RBD and hACE2. **(A)** The construction strategy of WT and variant RBD proteins. The RBD proteins were expressed in HEK293T cells by fusion of human IgG Fc tag in the form of secretion. **(B)** SDS-PAGE gel images of purified RBD proteins. **(C)** The ability of different variant RBDs to bind to hACE2 was test by ELISA. ELISA plate was coated with hACE2 protein (2 μg/ml). The fitted curves and half maximal effective concentration (EC50) were created using Graphpad software (version 8.0).

To determine the affinity between different variant RBD and hACE2, five RBD proteins (WT, Alpha, Beta, Delta, and Lambda) were fused with human IgG Fc and expressed in HEK293T cells (**Figure 2A**). SDS-PAGE gel electrophoresis showed that all RBD proteins were expressed in dimer form and the purity was more than 95% (**Figure 2B**). The affinity between different variant RBD and hACE2 receptors was measured by enzyme linked immunosorbent assays (ELISA). The results showed that the binding affinity of different variant RBDs to hACE2 receptors (Alpha: EC50 = 18.65 ng/ml; Beta: EC50 = 7.53 ng/ml; Delta: EC50 = 16.02 ng/ml; Lambda: EC50 = 27.19 ng/ml) were increased to varying degrees compared with that of WT RBD (WT: EC50 = 27.35 ng/ml) (**Figure 2C**). Particularly, Beta RBD has the highest affinity to hACE2 (3.6-fold compared to WT) among these variants.

### Beta and Multivalent RBD Antigens Induce Broad Spectrum Neutralization Antibodies

**Figure 3:**
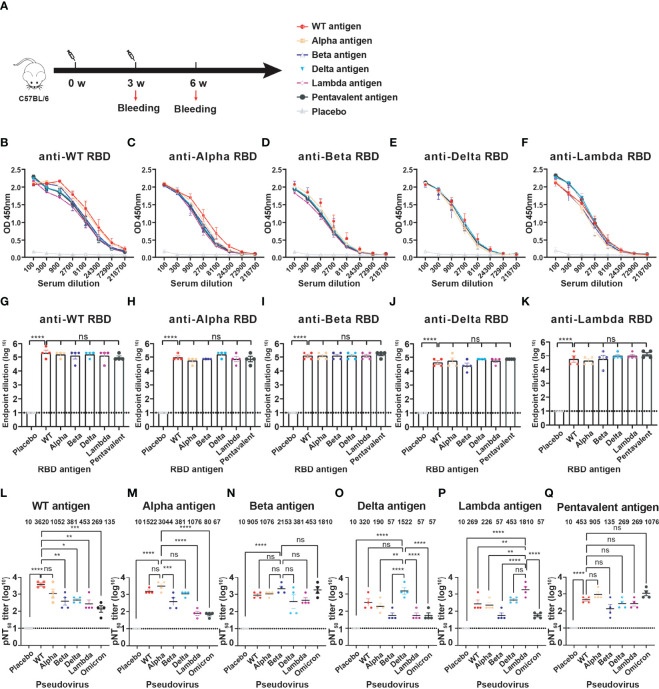
The cross-immunity evaluation of different variant RBD antigens. **(A)** The diagram of the immune program of monovalent and multivalent RBD antigens. Naive C57BL/6 mice (n = 4) were muscularly immunized and bled at the indicated time points. The immune dose is 10 μg per mouse of each variant RBD protein; Pentavalent RBD antigens was a mixture of 2 μg each variant RBD protein. Mice immunized with PBS defined as placebo control. **(B–F)** Detection of the specific IgG against different SARS-CoV-2 variant RBDs by ELISA. Serum samples were collected after 3-week boost immunization. A placebo formulation was given as the control. **(G–K)** The endpoint dilution titers of anti-different variant RBD antibodies induced by different monovalent and pentavalent RBD antigens. **(L–Q)** Neutralizing titers of different variant RBD antigens against different variant pseudoviruses. The data are shown as the mean ± SEM. The dotted horizontal lines indicate the limits of quantification for endpoint dilution and NT_50_ titers. P values were determined by one-way ANOVA (ns, p > 0.05; *p < 0.05; **p < 0.01; ***p < 0.001; ****p < 0.0001).

We prepared five RBD proteins (WT, Alpha, Beta, Delta, Lambda) and immunized mice to obtain antiserums for evaluation of the cross-immune reaction against different SARS-CoV-2 variants. Naive C57BL/6 mice (n=4) were immunized intramuscularly with monovalent or multivalent RBD antigens pre-bound to Alum-adjuvant. The priming and booster vaccinations were spaced at 3 weeks (**Figure 3A**). The immune dose was 10 μg each variant RBD protein per mouse for monovalent RBD antigen. Pentavalent RBD antigens were a 10 μg RBD protein mixture including each 2 μg variant RBD proteins. Blood samples were collected, and antiserum was separated at 3 weeks after the boost immunization. To detect the anti-RBD IgG antibody titers, the ELISA plates were coated with six different RBD protein respectively (WT, Alpha, Beta, Delta, Lambda, or Omicron). The OD value of each dilution is present in **Figures 3B–F**. In addition, there was no significant difference in the endpoint dilution of serum (IgG titers, ~ 10^5^) among different monovalent and pentavalent RBD antigen-induced antibodies against different variant RBD proteins (**Figures 3G–K**), which might be attributable to the whole structures of RBDs from all the variants were not significantly changed compared to that of WT. Furthermore, the neutralization titers (NT_50_) of the sera were measured using HIV-based pseudovirus neutralization assays. We observed that WT RBD antigen-induced antibodies showed a lower neutralizing activity against Beta, Delta, Lambda, and even Omicron pseudoviruses compared to neutralization against itself (**Figure 3L**). Immunization with WT RBD induced 26.8-fold lower neutralization titers against the Omicron pseudovirus compared to WT pseudovirus, indicating that the WT strain vaccine has lower cross-protective immunity against the Omicron variant. Similarly, Alpha (**Figure 3M**), Delta (**Figure 3O**), and Lambda (**Figure 3P**) RBD-induced antibodies exhibited high neutralizing titers against itself, but not against the Omicron variant. In contrast, Beta RBD antigen induced high neutralization antibodies against the Omicron variant (**Figure 3N**). Moreover, Beta RBD antigen (**Figure 3N**) and multivalent RBD antigen (**Figure 3Q**) induced broader cross-neutralization antibodies than other variant RBD antigens.

## Discussion

The SARS‐CoV‐2 variants have brought new challenges to the prevention and control of COVID‐19 (42, 43). There is an urgent need for a universal vaccine that could protect people from multiple COVID-19 variants. He *et al.* developed a SARS-CoV-2 bivalent recombinant vaccine (WT and Beta) targeting the S1 protein, which induces neutralizing antibodies against both SARS-CoV-2 variants and wild-type of the virus (44). Another study showed that the SARS-CoV-2 mRNA bivalent vaccine (WT and Beta) produced a broad-spectrum neutralization response against WT, Beta, CAL.20C, and P1 variants (45). Here, we expressed the five different RBD proteins (WT, Alpha, Beta, Delta, Lambda) and evaluated their cross-neutralizing activity against different pseudoviruses. The results demonstrate that Beta RBD antigen induces broader cross-neutralization antibodies against WT, Alpha, Beta, Delta, Lambda, and Omicron variant pseudoviruses compared with other monovalent RBD antigens, which is supported by the observation that rVSV-S Beta elicits cross-reactive neutralizing antibodies against WT strain, Alpha, and Beta variants (46).

We noticed that the multivalent RBD antigen induces broader cross-neutralization antibodies and higher neutralization titers against Omicron compared to the monovalent antigens. This indicates that there might be conserved neutralizing epitopes between different RBD variants, and more antibodies against conserved neutralizing epitopes were generated after repeated immunization with RBD multivalent antigens. There were similar cases of flu vaccines; the chimeric influenza HA vaccine contains different HA heads and the same HA stem, and more antibodies against the stem region can be produced after multiple immunizations (47–50).

Current studies suggest that the Omicron variant vaccine is not the best choice against the Omicron variant. It does not produce high neutralization titer and broad-spectrum neutralization ability against itself and other variants (51, 52). Interestingly, among these monovalent RBD antigens, only Beta RBD antigen induces high neuralization titers against Omicron, which might be attributable to the similar mutation sites (**Figure 1A**) and antigen sequences (**Figure 1B**) of Beta and Omicron RBDs.

In summary, our results showed that the cross-protective immunity of WT strain SARS-CoV-2 RBD antigen against the currently circulating variants was significantly reduced. In contrast, Beta RBD and multivalent RBD antigens both induced broad spectrum neutralization antibodies compared with other monovalent RBD antigens. Therefore, it is necessary to update or develop new COVID-19 vaccines. This study provides a reference for vaccine strain selection and universal COVID-19 vaccine design to fight the constant emergence of new SARS-CoV-2 variants.

## Data Availability Statement

The original contributions presented in the study are included in the article/supplementary material. Further inquiries can be directed to the corresponding authors.

## Ethics Statement

The animal study was reviewed and approved by The Experimental Animal Ethics and Welfare Committee of IMCAS.

## Author Contributions

WL and LS initiated the project and supervised the project. LS, WL, and WS designed the experiments, analyzed the data, and wrote the paper. WS performed the experiments; LH, HL, and WF helped with some experiments. GC and LY helped analyze the data and revised the manuscript. All authors contributed to the article and approved the submitted version.

## Funding

This work was supported by grants from the Strategic Priority Research Program of Chinese Academy of Sciences (XDB29010000).

## Conflict of Interest

The authors declare that the research was conducted in the absence of any commercial or financial relationships that could be construed as a potential conflict of interest.

## Publisher’s Note

All claims expressed in this article are solely those of the authors and do not necessarily represent those of their affiliated organizations, or those of the publisher, the editors and the reviewers. Any product that may be evaluated in this article, or claim that may be made by its manufacturer, is not guaranteed or endorsed by the publisher.
